# 

**Published:** 1886-11

**Authors:** 


### *NEW YORK STATE MEDICAL ASSOCIATION.*

This association will hold its third annual meeting Nov. 16th, 17th and 18th, at Lyric Hall, Sixth ave. between Forty-first and Forty-second street, New York City. The too former meetings have been of great scientific value, and the source of pleasure and profit to all who have attended. The programme for this year is just out and is almost up to those of past years, although we must confess there is a certain disappointment in the papers to be presented by New York men. From abroad, however, and especially from Western New York, there appear the names of those who will do the association credit. Among those from other states we notice the names of Prof. Charles T. Parkes of Chicago, Dr. Chas. B. Nancrede of Pennslyvania, Dr. J. B. Hamilton, Surgeon-General of the Marine Hospital service and Prof. James Tyson, of Philadelphia, who will discuss the paper of William Lusk, on Eclampsia. Among those from Western New York we finds the names of Prof. J. B. Andrews of Buffalo, who will report an interesting care of the Morphine and Cocaine habit. Prof. W. S. Tremaine will read a paper on "Gun Shot Wounds of the Abdomen." One of the features of the second day, morning session, will be an address on Foresnic medicine, by Prof. Simeon T. Clark, of the Niagara University, the orator of Western New York.

Prof. John Cronyn, of Niagara University, will discuss the paper of Henry D. Didama on Pulmonary Tuberculosis. Prof. Alvin A. Hubbell will read a paper on Congenital Stenosis ot the nose. On the third day Prof. Chas. G. Stockton will deliver the address, taking for his subject Therapeutics. Papers will also be read by Drs. Moore and Honey, of Rochester. Those intending to read papers, who have not already notified the committee should do so at once, by addressing Dr. E. D. Ferguson, Troy, N. Y.

